# Metformin Reduces Hepatic Expression of SIRT3, the Mitochondrial Deacetylase Controlling Energy Metabolism

**DOI:** 10.1371/journal.pone.0049863

**Published:** 2012-11-16

**Authors:** Marcin Buler, Sanna-Mari Aatsinki, Valerio Izzi, Jukka Hakkola

**Affiliations:** 1 Department of Pharmacology and Toxicology, Institute of Biomedicine, University of Oulu, Oulu, Finland; 2 Center for Cell-Matrix Research and Biocenter Oulu, Department of Medical Biochemistry and Molecular Biology, University of Oulu, Oulu, Finland; Université Joseph Fourier, France

## Abstract

Metformin inhibits ATP production in mitochondria and this may be involved in the anti-hyperglycemic effects of the drug. Sirtuin 3 (SIRT3) is a mitochondrial protein deacetylase that regulates the function of the electron transport chain and maintains basal ATP yield. We hypothesized that metformin treatment could diminish mitochondrial ATP production through downregulation of SIRT3 expression. Glucagon and cAMP induced SIRT3 mRNA in mouse primary hepatocytes. Metformin prevented SIRT3 induction by glucagon. Moreover, metformin downregulated constitutive expression of SIRT3 in primary hepatocytes and in the liver *in vivo*. Estrogen related receptor alpha (ERRα) mediates regulation of *Sirt3* gene by peroxisome proliferator-activated receptor gamma coactivator 1-alpha (PGC-1α). ERRα mRNA expression was regulated in a similar manner as SIRT3 mRNA by glucagon, cAMP and metformin. However, a higher metformin concentration was required for downregulation of ERRα than SIRT3. ERRα siRNA attenuated PGC-1α mediated induction of SIRT3, but did not affect constitutive expression. Overexpression of the constitutively active form of AMP-activated protein kinase (AMPK) induced SIRT3 mRNA, indicating that the SIRT3 downregulation by metformin is not mediated by AMPK. Metformin reduced the hepatocyte ATP level. This effect was partially counteracted by SIRT3 overexpression. Furthermore, metformin decreased mitochondrial SIRT3 protein levels and this was associated with enhanced acetylation of several mitochondrial proteins. However, metformin increased mitochondrial mass in hepatocytes. Altogether, our results indicate that metformin attenuates mitochondrial expression of SIRT3 and suggest that this mechanism is involved in regulation of energy metabolism by metformin in the liver and may contribute to the therapeutic action of metformin.

## Introduction

Biguanide class drug metformin is one of the most prescribed drugs for treatment of type 2 diabetes worldwide. Metformin has been reported to have numerous cellular effects in multiple tissues, but the main anti-hyperglycemic effect is believed to be due to the suppression of hepatic glucose production [1]. AMP-activated protein kinase (AMPK) is activated by metformin and has been a strong candidate in mediating therapeutic effects of the drug [2]. However, a recent study showed that AMPK deficiency did not abolish the effects of metformin on hepatic glucose production, indicating that the role of AMPK is dispensable [3]. Furthermore, neither AMPK nor upstream kinase serine/threonine kinase 1 (LKB1) are direct targets of metformin [1], [4]. Indeed, it has been suggested that a decreased energy state and reduced intracellular ATP content are the primary mechanisms mediating metformin action on hepatic glucose production [3]. Metformin inhibits Complex I of the mitochondrial respiratory chain, but the exact mechanisms and pathways involved are unclear [5], [6].

The sirtuins (SIRT) are a family of nicotinamide adenine dinucleotide (NAD^+^) dependent deacetylase proteins with broad cellular functions including regulation of energy production, fatty acid metabolism and the cell cycle [7], [8]. SIRT3 is a crucial regulator of mitochondrial function, controlling global acetylation of the organelle. Enzymatic activity of SIRT3 induces activity of Complex I and promotes oxidative phosphorylation. Mitochondrial proteins of SIRT3 knockout mice are hyperacetylated and cellular ATP levels of such animals are reduced in high energy tissues in, for example, the liver, heart and kidneys [9]. Therefore, loss of SIRT3 appears to decrease mitochondrial substrate oxidation and results in more intensive glycolysis and higher extracellular lactate levels [10]. In line with these observations, SIRT3 knockout attenuates oxygen consumption, while overexpression increases it [11], [12]. The effect of SIRT3 deficiency on ATP production is further aggravated by fasting, underlying its reliance on NAD+[13].

Expression of SIRT3 in the liver is induced by peroxisome proliferator-activated receptor gamma coactivator 1-alpha (PGC-1α) through a mechanism involving estrogen-related receptor alpha (ERRα) [14]. Coactivator PGC-1α and nuclear receptor ERRα are transcriptional regulators controlling expression of genes involved in energy homeostasis, mitochondrial biogenesis, fatty acid oxidation and glucose metabolism [15], [16]. Both PGC-1α and ERRα are induced by fasting and consequently increase SIRT3 levels.

Mitochondrial function appears to be the key target of metformin, and reduction of ATP production may mediate the hepatic, anti-hyperglycemic action of the drug. Since SIRT3 has been shown to directly regulate mitochondrial function and enhance ATP production, we hypothesized that metformin could downregulate SIRT3 expression and/or activity. Therefore, we studied the effect of metformin on SIRT3 expression and function in mouse hepatocytes. We show that metformin downregulates SIRT3 expression and that this results in increased mitochondrial protein acetylation.

## Materials and Methods

### Materials

8-Bromo-cAMP (8-Br-cAMP), porcine glucagon, metformin and Fluoroshield with DAPI were purchased from Sigma-Aldrich (St. Louis, MO, USA). Permanox Lab-Tek™ Chamber Slides (#177445) were obtained from Thermo Fisher Scientific (Walthman, MA, USA), Mitotracker Green FM from Invitrogen (Carlsbad, CA, USA) and Any-kDa precast gels from Bio-Rad (Hercules, CA, USA).

### Preparation of Primary Hepatocytes and Cell Culture

Primary hepatocytes were isolated from male DBA/2 (OlaHsd) mice (Center for Experimental Animals, University of Oulu, Finland) aged 8 to 10 weeks and cultured as described previously [17]. The cultures were maintained for 24 hours before adenoviral infections or chemical treatments. 8-Br-cAMP, glucagon and metformin were all dissolved in water and the control samples were left untreated. Adenovirus infections and primary hepatocyte treatments were performed in serum-free William’s E medium. HepG2 cells were cultured in DMEM medium (Invitrogen) containing 10% (v/v) fetal calf serum, 100 U/ml of penicillin and 100 µg/ml streptomycin (Invitrogen) and 4500 mg/l glucose.

### Metformin in vivo Experiment

Fourteen male DBA/2 mice (Center for Experimental Animals, University of Oulu, Finland) aged 12–13 weeks were housed in standard conditions with 12-hour dark-light cycles. The mice had free access to drinking water and standard animal chow throughout the entire experiment. Mice in the treatment group (n = 7) were given metformin (Sigma-Aldrich) dissolved in vehicle (physiological saline), 300 mg/kg intragastrically (i.g.) (∼200 µl/mouse) once daily for 7 days. The control group (n = 7) received the same volume of vehicle. At the end of the experimental time period, mice were anesthetized with a solution containing fentanyl-fluanisone (Hypnorm®) and midazolam (Dormicum®) and killed by neck dislocation, after which livers were rapidly collected and snap-frozen in liquid nitrogen. All animal experiments were approved by the Finnish national committee of laboratory animal welfare.

### RNA Preparation and Quantitative RT-PCR

Total RNA was isolated using TRI-reagent (Molecular research center, Cincinnati, OH, USA) according to the manufacturer’s protocol and treated with DNase (Promega, Fitchburg, WI, USA). 1 µg of RNA was reverse transcribed to produce cDNA using p(dN)6 random primers (Roche, Basel, Switzerland) and M-MLV reverse transcriptase (Promega). The quantitative PCR reactions were performed using FastStart Universal SYBRGreen Master Mix or FastStart Universal Probe master mix (Roche). The primer sequences were as follows (Forward/Reverse in 5′ to 3′ order): 18S CGCCGCTAGAGGTGAAATTC/CCAGTCGGCATCGTTTATGG; SIRT3 GCTGACGACTTCGACGACG/TCGGTCAACAGGAGGTTGTCT; ERRα ATCTGCTGGTGGTTGAACCTG/AGAAGCCTGGGATGCTCTTG; SIRT1 ATCCCGGACTTCAGATCCCC/CAACATGAAAAAGGGCTTGGG; PPARα AGAGCCCCATCTGTCCTCTC/ACTGGTAGTCTGCAAAACCAAA. CYP17A1 was measured using a TaqMan™ probe set Mm00484041_g1 from Applied Biosystems (Carlsbad, CA, USA). Fluorescence values of the QPCR products were corrected with the fluorescence signals of the passive reference dye (ROX). The RNA levels of target genes were normalized against the 18S control levels using the comparative C_T_ (ΔΔC_T_) method.

### Measurement of Mitochondrial DNA

DNA was isolated from primary hepatocytes and liver tissue using phenol–chloroform extraction; 30 ng of DNA was used per qPCR reaction. The mitochondrial DNA level was measured with primers targeted for mouse mitochondrial *D-Loop* with SYBRGreen chemistry using the following primers (Forward/Reverse in 5′ to 3′ order): AATCTACCATCCTCCGTG/GACTAATGATTCTTCACCGT [18] and normalized against the nuclear DNA level measured with the above-mentioned 18S primers using the comparative C_T_ (ΔΔC_T_) method. DNA from HepG2 hepatoma cells was isolated with QIAamp DNA Mini Kit (Qiagen, Venlo, N.V., USA); 100 ng of DNA was used per qPCR reaction. The mitochondrial DNA level was measured with qPCR using FAM/TAMRA probes for *ND1* gene and normalized against nuclear DNA levels of *BNP* gene as above. *ND1* primers: CCCTAAAACCCGCCACATC/GAGCGATGGTGAGAGCTAAGGT, probe: CCATCACCCTCTACATCACCGCCC; *BNP* primers: CAGGAGCAGCGCCAACCAT//CAGGGATGTCTGCTCCACCT, probe: GTGCAGGGCAAACTGTCGGAG [19].

### Protein Isolation and Immunoblotting

Mitochondria were isolated with a Qproteome Mitochondria Isolation Kit (Qiagen) according to the manufacturer’s protocols. Whole cell lysate fraction was prepared as described previously [20]. Samples were separated on SDS-polyacrylamide gel, transferred to a polyvinylidene fluoride membrane (Millipore, Billerica, MA, USA) and incubated with appropriate primary antibody in 5% skimmed milk in Tris buffered saline with 0.1% Tween, followed by HRP conjugated IgG (1∶20000). The antibodies were as follows: SIRT3 (ab86671, Abcam, Cambridge, UK); acetyl lysine (ab21623, Abcam); VDAC1 (ab14734, Abcam), MTCO1 (ab14705, Abcam), GAPDH (MAB374, Millipore, Billerica, MA, USA); β-actin (A1978, Sigma-Aldrich). After washing, the immunoreactive bands were visualized with Chemiluminescent Peroxidase Substrate (CPS) 1 reaction (Sigma-Aldrich) using Quantity One software (Bio-Rad).

### Plasmids, Transient Transfection and Adenoviruses

A plasmid with Flag tagged SIRT3 was obtained from Addgene (www.addgene.org) (Addgene plasmid 13814) and has been described earlier [21]. HepG2 cells in 96-well plates were transfected with 500 ng of SIRT3 plasmid or with empty pcDNA3 control plasmid in a volume of 125 µl using Fugene HD (Roche) according to the manufactureŕs protocol 24 hours before metformin treatment. The recombinant adenoviruses expressing green fluorescent protein (GFP-Ad), human constitutively active AMPKα1 subunit containing amino acids 1–321 (AMPK-Ad) and PGC-1α (PGC-1α-Ad) have been described previously [17], [22]. LacZ-Ad control virus was kindly provided by Dr. Heikki Ruskoaho (University of Oulu).

### siRNA Experiment

Mouse primary hepatocytes were transfected with Lipofectamine 2000 (Invitrogen) using 150 pmol/well of the following siRNAs (Sigma-Aldrich): siERRα 5′GAGCAUCCCAGGCUUCUCC(dT)(dT) [23]; scramble 5′AAGCUUCAUAAGGCGCAUAGC(dT)(dT), according to the manufacturer’s instructions for cells in 6-well plates. After a 5 hour incubation period, the transfection medium was replaced by Williams E medium (Sigma-Aldrich) containing either PGC-1α expressing adenovirus (PGC-1α-Ad) or LacZ-Ad used as a control at MOI 0.1. After 48 hours post-infection, the cells were collected for total RNA isolation. The efficiency of the knockdown was tested by measuring ERRα mRNA by qPCR.

### ATP and ADP Measurements

Cells were cultured in 96-well plates for 24 hours before treatment. At indicated time points, medium was removed, cells washed with PBS and ATP/ADP levels measured with EnzyLight ADP/ATP Ratio Assay Kit (BioAssay Systems, Hayward, CA, USA) according to the manufacturer’s protocol.

### Fluorescense Microscopy

Cells were cultured on Permanox Lab-Tek 8-well chamber slides with metformin for 48 hours, fixed with 4% PFA for 15 min and treated with 5 mg/ml of BSA (Invitrogen) for 1 hour, incubated either with anti-HA-Tag (6E2) (1∶100, #2367, Cell Signaling Technology, Boston, MA, USA) or anti-MTCO1 (1∶100, ab14705, Abcam) antibody for 1 hour in PBS with 0.05% Triton × (Sigma-Aldrich), followed by 1-hour staining with Alexa 488 goat anti-mouse (H+L) (A-11001, Invitrogen,) antibody (1∶1000). Alternatively, cells were incubated for 1 hour in medium supplemented with Mitotracker Green FM (100 nM) and washed extensively with PBS (cells were not fixed at any stage). After the treatment, samples were mounted with Fluoroshield with DAPI (Sigma-Aldrich) and observed under an Olympus FluoView FV1000 confocal microscope.

Confocal fluorescence imaging: Mouse primary hepatocytes: DAPI, 405 nm transmissivity 6.5%, emission wavelength 461 nm; Alexa 488 goat anti-mouse (H+L), 488 nm transmissivity 5%, emission wavelength 520 nm. HepG2 cells: DAPI, 405 nm transmissivity 10%, emission wavelength 461 nm; Mitotracker Green FM, 488 nm transmissivity 7.7%, emission wavelength 520 nm. Identical settings were maintained throughout the respective experiments.

### Flow Cytometry (FACS)

HepG2 cells were cultured in 100 mm dishes with 3 mM metformin for 48 hours, washed with PBS and incubated with Mitotracker Green FM (100 nM) for 1 hour, collected and analyzed using a FACSCalibur cytometer running CellQuest Pro software. Unstained HepG2 cells were used to set up acquisition parameters, and 1×10^6^ events were collected for each sample.

### Statistical Analysis

Statistical data analysis was done using GraphPad Prism Software. The comparison of means of two groups was done by Student’s *t*-test, whereas multiple groups were compared by one-way ANOVA followed by Bonferroni post hoc test. Differences were considered statistically significant when P<0.05.

## Results

### Metformin Affects SIRT3 Expression

**Figure 1:**
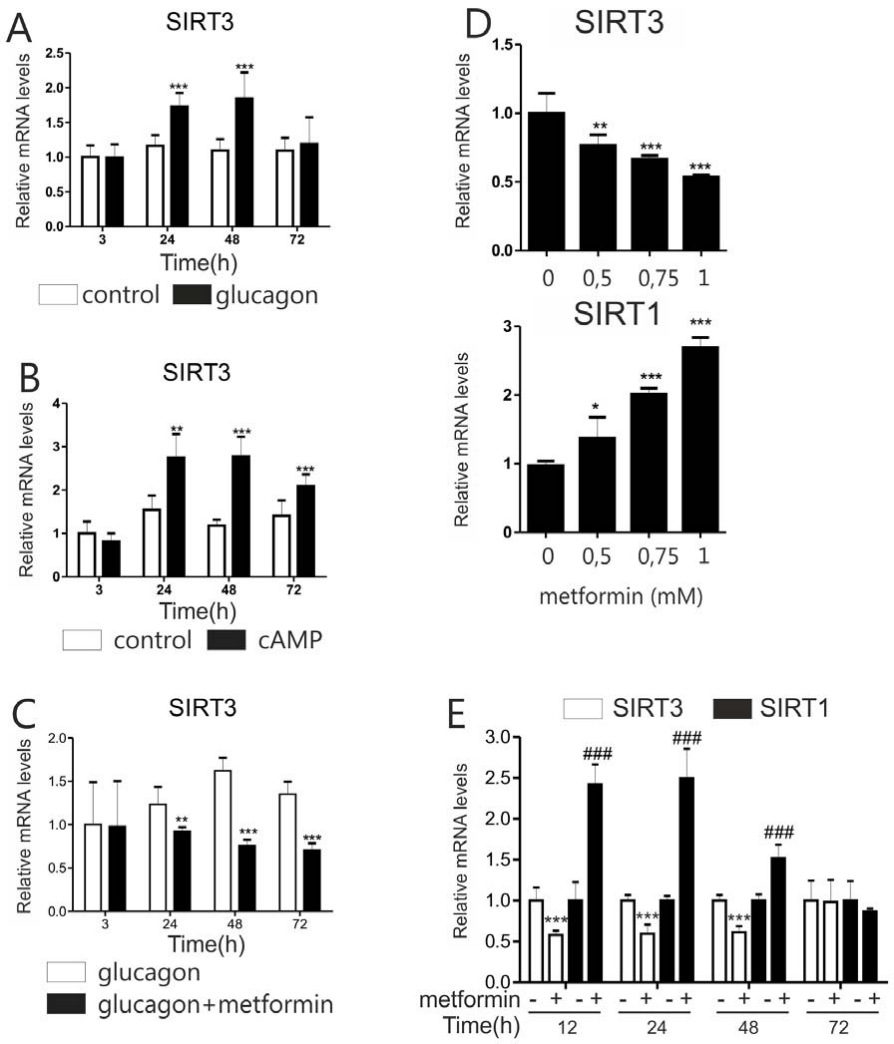
SIRT3 expression is regulated by glucagon, cAMP and metformin. Effect of **A)** glucagon (5 µg/ml), **B)** cAMP (50 µM 8-Br-cAMP) and **C)** glucagon (5 µg/ml) combined with metformin (1 mM) on SIRT3 expression in mouse primary hepatocytes. Cells were collected at indicated time points and expression of SIRT3 mRNA measured with qPCR. **D)** Mouse primary hepatocytes were treated with metformin as indicated and collected after 24 hours. mRNA levels were measured with qPCR. In panels A-D, the data are presented as mean+SD (n = 3). Statistical significance to control samples *P<0.05, **P<0.01, *** P<0.001 (ANOVA, Bonferroni post hoc test). **E)** Mouse primary hepatocytes were treated with 1 mM metformin, collected as indicated and the mRNA levels were measured with qPCR. The values are presented as mean+SD (n = 4) and relative to the untreated samples at each time point. Statistical significance for SIRT3 and SIRT1 was calculated independently. SIRT3 *** P<0.001; SIRT1 ### P<0.001 (ANOVA, Bonferroni post hoc test).

Fasting and fasting-inducible coactivator PGC-1α induce expression of SIRT3 [13], [14]. For that reason, we investigated if glucagon, a hormone upregulating PGC-1α expression and induced by fasting, affects SIRT3. Mouse primary hepatocytes were treated with glucagon (5 µg/ml) and SIRT3 mRNA expression was measured at several time points up to 72 hours. Glucagon significantly induced SIRT3 mRNA after 24 and 48 hours (Fig. 1a). Glucagon’s secondary messenger, cAMP (50 µM 8-Br-cAMP), had a similar effect, however, statistically significant induction was detected even after 72 hour (Fig. 1b).

The antidiabetic drug metformin attenuates induction of gluconeogenic genes such as phosphoenolpyruvate carboxykinase 1 by glucagon [24]. We investigated if metformin affects *Sirt3* regulation by glucagon in a similar manner. Primary hepatocytes were cotreated with glucagon and 1 mM metformin and SIRT3 mRNA expression was measured. Metformin severely impaired SIRT3 induction by glucagon (Fig 1c). Subsequently, we measured whether metformin alone affects SIRT3 mRNA level. Indeed, its expression was reduced by the treatment in a dose-dependent manner and 1 mM metformin downregulated SIRT3 mRNA by about 50% (Fig. 1d). In contrast, expression of SIRT1, another member of the Sirtuin family, was induced 2.7-fold by the treatment (Fig. 1d). In a time course experiment, the opposite regulation of the two Sirtuins was observed from 12 to 48 hours after metformin treatment (Fig. 1e).

### Metformin Downregulates ERRα

**Figure 2:**
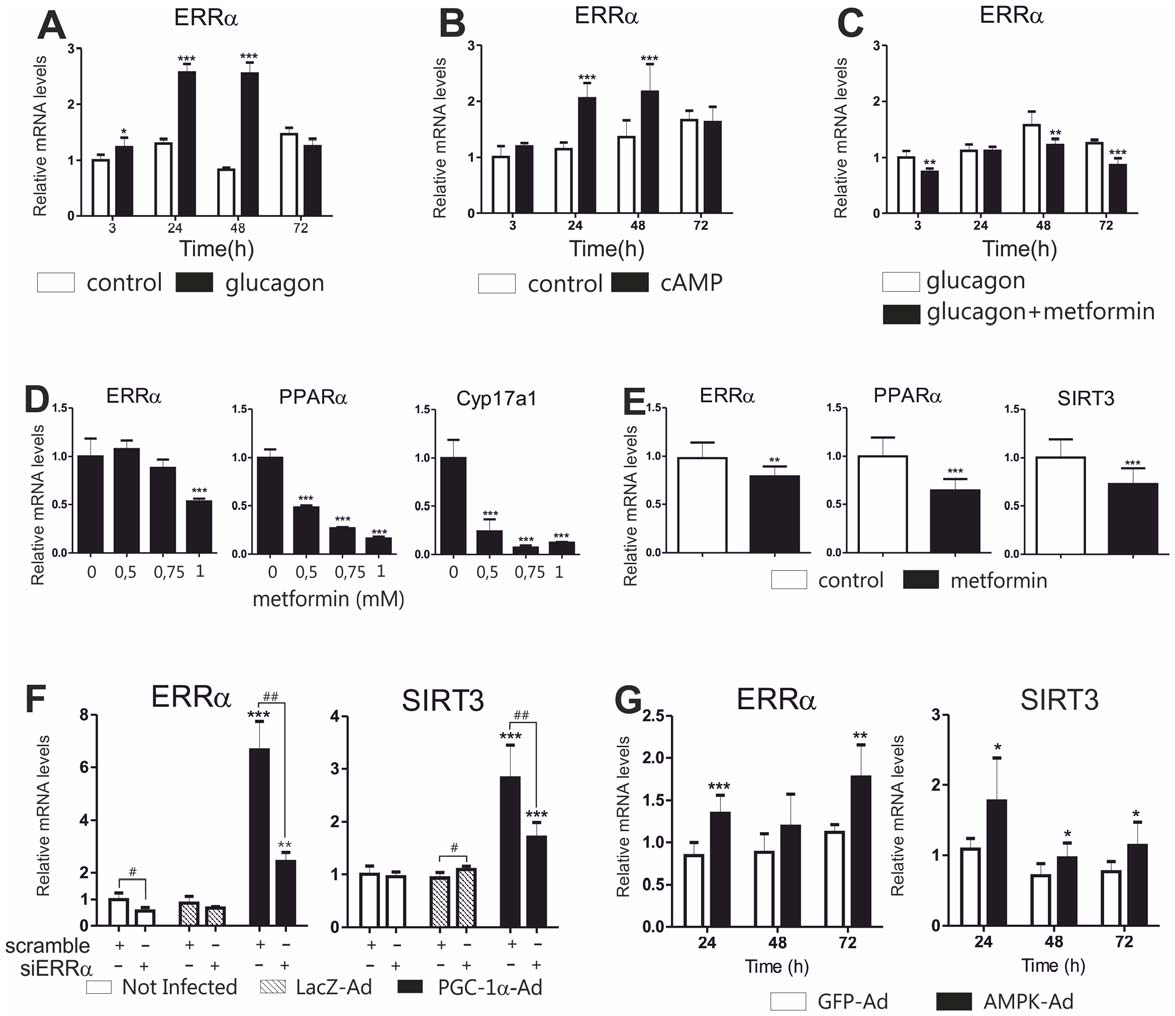
Involvement of ERRα in regulation of SIRT3 by metformin. Effect of **A)** glucagon (5 µg/ml), **B)** cAMP (50 µM 8-Br-cAMP) and **C)** glucagon (5 µg/ml) combined with metformin (1 mM) on ERRα expression in mouse primary hepatocytes. Cells were collected at indicated time points and expression of ERRα mRNA measured with qPCR. **D)** Mouse primary hepatocytes were treated with metformin as indicated, collected after 24 hours and mRNAs were measured with qPCR. In panels A-D, the data are presented as mean+SD (n = 3). Statistical significance to control samples **P<0.01, *** P<0.001 (ANOVA, Bonferroni post hoc test). **E)** Effect of metformin treatment on ERRα and SIRT3 mRNA expression in the liver. Mice (n = 7) were treated for seven days with metformin 300 mg/kg i.g. once daily for 7 days. Control animals received physiological saline only. Statistical significance to control samples **P<0.01, *** P<0.001 (Student two tailed t-test). **F)** Effect of ERRα siRNA on SIRT3 expression in mouse primary hepatocytes. Adenoviruses were used at MOI 0.1. Values represent mean+SD, n = 3. Data represent fold change relative to the samples treated with scramble siRNA and not infected with any of the adenoviruses. A statistically significant effect of the adenovirus treatment is indicated with asterisks, ** P<0.01, ***P<0.001 (ANOVA, Bonferroni post hoc test). A statistically significant effect of the ERRα siRNA treatment compared to the scramble siRNA is indicated with hash marks, # P<0.05, ## P<0.01 (Student two tailed t-test). **G)** Effect of AMPK on SIRT3 and ERRα expression. Mouse primary hepatocytes were infected with AMPK-Ad or GFP-Ad (MOI = 2) or left untreated. Cells were collected at indicated time points and mRNA expression measured with qPCR. Values represent mean+SD, n = 4. Data represent fold change compared to untreated controls at a given time point. * P<0.05, ** P<0.01, ***P<0.001, statistical significance compared to GFP-Ad (Student two tailed t-test).

ERRα is a nuclear receptor induced by fasting [25], and controls transcription of *Sirt3* gene [14]. Consistently with these observations, ERRα levels were elevated in cells treated with glucagon and cAMP (Fig. 2a, b). On the other hand, metformin attenuated induction of ERRα mRNA by glucagon (Fig. 2c). Moreover, treatment of primary hepatocytes with metformin alone reduced ERRα (Fig. 2d). To further study the significance of ERRα reduction, the expression of two established ERRα target genes, *Pparα* [26] and *Cyp17a1* [27], was measured. Indeed, PPARα and CYP17A1 mRNAs were downregulated by metformin; however, both genes, as well as SIRT3, were affected by lower concentrations of metformin than ERRα (Fig. 2d, Fig. 1d).

In the next phase, we performed an *in vivo* experiment. Mice were treated with metformin (300 mg/kg i.g. once a day) for seven days and hepatic mRNA expression was compared to vehicle-treated controls (physiological saline). A relatively high dose of metformin was used because of earlier reports suggesting that high doses of i.g. metformin are necessary in rodents to reach plasma concentrations similar to those found in patients [28] and also because a similar dose was used in a previous study to demonstrate metformin’s effect on blood glucose in AMPK-deficient mice [3]. ERRα, SIRT3 and PPARα mRNAs were all statistically significantly downregulated by metformin (Fig. 2e). The contribution of ERRα reduction to SIRT3 downregulation by metformin was further studied by knockdown of ERRα in primary hepatocytes. ERRα siRNA did not affect constitutive expression of SIRT3. In contrast, PGC-1α-mediated induction of SIRT3 was significantly attenuated by ERRα knockdown (Fig. 2f).

### Downregulation of SIRT3 and ERRα by Metformin does not Involve AMPK

AMPK is activated by metformin and mediates many effects of the drug. Therefore, we investigated whether AMPK would affect expression of SIRT3 and ERRα. Mouse primary hepatocytes were transduced with adenovirus bearing a sequence of constitutively active AMPK (AMPK-Ad). In contrast to metformin, AMPK-Ad induced both SIRT3 and ERRα, suggesting that the downregulation of these genes by metformin is not mediated through AMPK (Fig. 2g).

### Metformin Inhibits Mitochondrial ATP Production and SIRT3 Expression and Function

**Figure 3:**
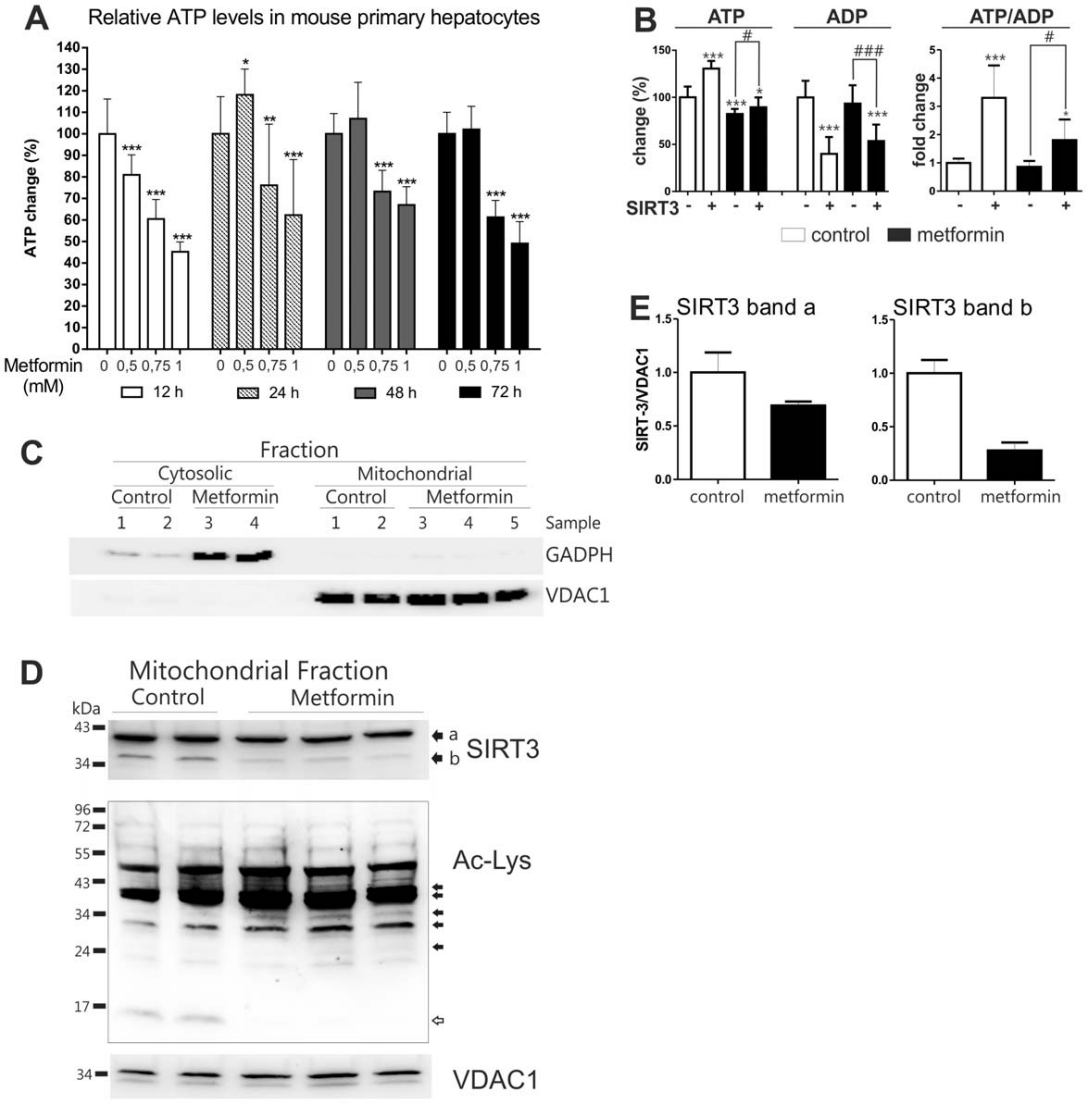
Metformin reduces ATP synthesis and mitochondrial SIRT3 expression and affects mitochondrial acetylation. **A)** Effect of metformin on cellular ATP level. Mouse primary hepatocytes were treated with metformin and ATP levels were measured at indicated time points. Data are presented as mean+SD (n = 12). Statistical significance to 0 mM metformin samples at each time point *P<0.05, ** P<0.01,*** P<0.001 (ANOVA, Bonferroni post hoc test). **B)** HepG2 cells were transfected either with SIRT3 plasmid or pcDNA3 control plasmid for 24 hours and treated with 3 mM metformin for an additional 24 hours. Thereafter the cells were collected, ATP and ADP measured and the ATP/ADP ratio calculated. In each case, values are presented relative to untreated samples (without SIRT3). Data is presented as mean+SD (n = 12). Statistical significance to control samples (without SIRT3) *P<0.05, *** P<0.001; statistically significant differences of pcDNA3 and SIRT3 samples treated with metformin #P<0.05, ### P<0.001, (ANOVA, Bonferroni post hoc test). **C)** Evaluation of mitochondrial fraction integrity. Mouse primary hepatocytes were treated with 1 mM metformin for 72 hours. Cytosolic and mitochondrial fractions from the same samples were isolated and subjected to Western blot analysis. **D)** Metformin affects SIRT3 expression and mitochondrial protein acetylation. Western blot analysis of the same mitochondrial fractions as in fig. 3C. Upper panel: immunostaining with anti-SIRT3 antibody, middle panel: immunostaining with anti-acetyl lysine antibody and lower panel: VDAC1 was detected as a loading control. Closed arrows indicate proteins hyperacetylated by metformin treatment, an open arrow indicates a protein deacetylated by metformin treatment. **E)** Quantitation of SIRT3 intensity in Western blot shown in fig. 3D (upper panel). Bands a and b were quantified separately and normalized to VDAC1.

A study by Foretz et al. [3] suggested that metformin reduces ATP synthesis in hepatocytes independently of the AMPK/LKB1 pathway. To study the effect of the drug on energy metabolism, we treated mouse primary hepatocytes with different doses of metformin for up to 72 hours and measured intracellular ATP content. In line with earlier reports, ATP levels were generally reduced by the treatment (Fig. 3a). Furthermore, treatment of HepG2 hepatoma cells with 3 mM metformin impaired ATP synthesis, while overexpression of SIRT3 increased ATP content in a dose-dependent manner and partially counteracted metformin effect (Fig. 3b). Because HepG2 cells do not express OCT1 transporter which is important for metformin uptake, a higher concentration of metformin was used in HepG2 cultures than with primary hepatocytes [29]. SIRT3 overexpression also significantly decreased ADP and increased the ATP/ADP ratio in HepG2 cells (Fig. 3b). Metformin treatment alone did not change the ATP/ADP ratio to a statistically significant level, but upregulation of the ATP/ADP ratio by SIRT3 was clearly reduced by metformin (Fig. 3b).

Consequently, we investigated whether metformin affects mitochondrial SIRT3 content. Mitochondrial fraction from metformin-treated and control hepatocytes was isolated and subjected to western blotting. We evaluated the quality of the fractionation method by measuring GAPDH levels in our samples. GAPDH is mainly a cytosolic protein, but also a transcriptional factor present in the nucleus [30], [31], while VDAC1 is an exclusively mitochondrial protein. GAPDH was hardly detectable in the mitochondrial fraction, even at the highest sensitivity settings, indicating a negligible cross-fraction contamination (Fig. 3c). Next, we measured the level of SIRT3 in the mitochondrial fraction. A 36 kDa anti-SIRT3 immunoreactive band was detected, which is similar to the one reported by the antibody manufacturer (Abcam, Cambridge, UK) (Fig. 3d). However, an additional anti-SIRT3 immunoreactive protein (ca. 42 kDa) was detected (Fig. 3d). There is some controversy in the literature concerning the exact size of the SIRT3 isoforms [32]–[34]. The two anti-SIRT3 immunoreactive proteins detected in the isolated mitochondria may correspond to preprocessed (ca. 42 kDa) and processed (ca. 36 kDa) SIRT3. Regardless of their sizes, expression of both isoforms was reduced by metformin (Fig. 3e). Because the loss of SIRT3 leads to mitochondrial global hyperacetylation [35], we studied whether metformin has a similar effect. For that reason, the western blot used to study mitochondrial SIRT3 expression was restained with acetylated lysine antibody. Indeed, metformin increased acetylation of several mitochondrial proteins in primary hepatocytes (Fig. 3d). However, there was also one protein that was deacetylated after metformin treatment (Fig. 3d).

### Metformin Induces Mitochondrial Biogenesis in Hepatocytes

**Figure 4:**
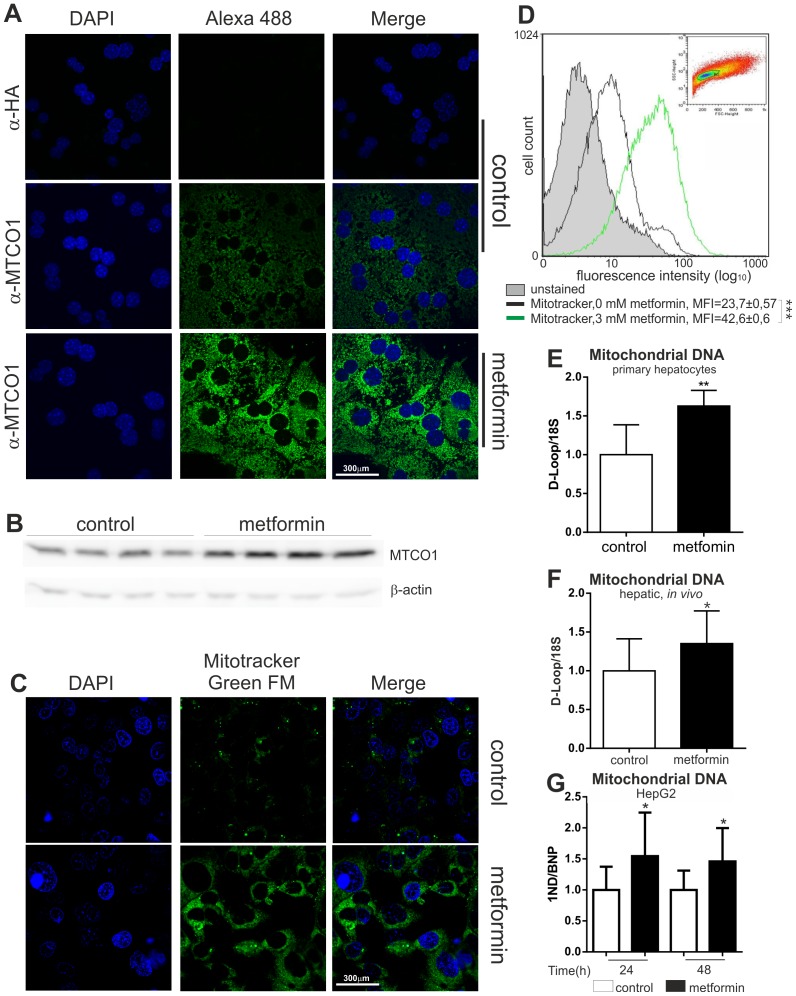
Effect of metformin on hepatic mitochondrial biogenesis. **A)** Mouse primary hepatocytes were cultured on permanox Lab-Tek 8-well chamber slides with 1 mM metformin for 48 hours. Cells were fixed with 4% PFA, immunostained as described and mounted with Fluoroshield with DAPI. MTCO1 stained green, cell nuclei blue. Representative images are shown. **B)** Western blot of whole cell lysates from mouse primary hepatocytes incubated with or without metformin for 48 hours. Upper panel: immunostaining with anti-MTCO1; lower panel: β-actin was detected as a loading control. **C)** HepG2 cells were cultured on permanox Lab-Tek 8-well chamber slides with 3 mM metformin for 48 hours, incubated with Mitotracker Green FM (100 nM) for 1 hour and mounted with Fluoroshield with DAPI. Mitochondria stained green, cell nuclei blue. Representative images are shown. **D)** Mitochondrial mass of HepG2 cells, treated with 3 mM metformin for 48 hours, was measured by FACS and compared with untreated cells by fluorescence levels upon staining with MitoTracker Green FM. A representative experiment is shown, which was repeated two additional times with similar results. MFI (Mean Fluorescence Intensity), ANOVA, Neuman–Keuls post-hoc test, *** P<0.001. Relative mitochondrial DNA level **E)** in mouse primary hepatocytes (n = 6) treated with metformin for 48 hours, **F)** in livers of mice treated with metformin for 7 days (300 mg/kg) (control n = 6, metformin n = 7), **G)** in HepG2 cells (n = 3) treated with 3 mM metformin for either 24 or 48 hours. In panels E-G, * P<0.05, ** P<0.01 (Student one tailed t-test).

Metformin has been reported to induce mitochondrial biogenesis, at least in certain cell types [36]. Interestingly, both ERRα and SIRT3 have been shown to be involved in mitochondrial biogenesis [14], [37], but our results indicated that metformin reduces expression of these factors. This discrepancy prompted us to investigate the possible effect of metformin on mitochondrial content in hepatocytes. Primary hepatocytes were treated with 1 mM metformin for 48 hours and subjected to immunofluorescence microscopy. As seen on representative microscope slides, metformin induced immunoreactivity with antibody against mitochondrially encoded cytochrome c oxidase I (MTCO1) protein (Fig. 4a). Similar results were observed in HepG2 cells treated with 3 mM metformin for 48 hours (data not shown). Furthermore, an increase in the amount of MTCO1 protein was detected by immunoblotting in whole cell lysates from mouse primary hepatocytes treated with metformin for 24 (data not shown) and 48 hours (Fig. 4b). MTCO1 is a specific mitochondrial protein coded by the organelle genome; however, its induction does not necessarily reflect increased mitochondrial biogenesis and could also be the result of a specific gene regulation event. Therefore, we used Mitotracker Green FM to visualize whole mitochondria in HepG2 cells treated with 3 mM metformin for 48 hours. As seen on representative microscope slides (Fig. 4c) and quantitatively by FACS measurement (Fig. 4d), metformin induced mitochondrial mass. In agreement with these results, the mitochondrial DNA/nuclear DNA ratio was induced by metformin in mouse primary hepatocytes (Fig. 4e) after 48-hour treatment. Hepatic mitochondrial DNA was also modestly, but significantly, increased *in vivo* after mice had been treated with metformin for 7 days (Fig. 4f). A similar effect was also observed in HepG2 cells after 24 and 48 hours (Fig. 4g).

## Discussion

In the current study, we show that metformin downregulates SIRT3 expression in the liver. Metformin prevented induction of SIRT3 by glucagon and also reduced SIRT3 expression *per se* in mouse primary hepatocytes. A similar effect was observed *in vivo.* By contrast, but in agreement with previous studies, SIRT1 was upregulated by the drug [38]. These findings indicate that metformin distinctively regulates expression of different Sirtuin family members.

In agreement with previous studies on cultured cells and mouse livers *in vivo* [3], [5], we observed that metformin inhibits ATP synthesis. At the same time, SIRT3 protein expression was decreased in the hepatocyte mitochondria. Because of SIRT3 enzymatic function as a mitochondrial deacetylase, it’s lower expression should lead to stronger mitochondrial acetylation [9], [35]. Indeed, acetylation of several mitochondrial proteins was enhanced, suggesting that metformin can regulate mitochondrial function through a SIRT3-mediated mechanism, and this may contribute to a reduced intracellular ATP level. This was further supported by the fact that overexpression of SIRT3 in HepG2 attenuated the inhibitory effect of metformin on ATP synthesis. Furthermore, SIRT3 overexpression increased the ATP/ADP ratio. These findings are in agreement with earlier studies reporting mitochondrial hyperacetylation and reduced ATP levels in *Sirt3* null mice [9], [13]. Several studies have suggested that metformin acts on intact cells, but has no influence on isolated mitochondria [6], [39] indicating an indirect effect on mitochondrial function through an unknown signaling pathway. On the other hand, direct inhibition of Complex I by metformin has been reported [5]. Our results do not exclude the possibility of a direct effect of metformin on Complex I; however, SIRT3 is a strong candidate for the potential mediator of the indirect effects.

Reduction of ATP production may play a key role in inhibition of hepatic glucose production by metformin [3]. Therefore, downregulation of SIRT3 is expected to make an important contribution to the therapeutic action of metformin. However, in contrast to this hypothesis of the beneficial effect of SIRT3 downregulation, a recent study reported on the downregulation of SIRT3 expression by a high-fat diet and acceleration of the development of metabolic syndrome by SIRT3 knockout [40]. Noteworthy, the downregulation of SIRT3 by a high-fat diet was associated with reduced expression of PGC-1α, which is a major regulator of SIRT3 expression [14], [40]. Intriguingly, however, hepatic expression of PGC-1α is induced in several mouse models of diabetes [41], suggesting that in the actual diabetic state, hepatic metabolic regulation differs from the high-fat diet model mentioned above. Indeed, increased glucose production in livers of diabetic patients seems to involve abnormal activation of mechanisms physiologically activated by fasting, including PGC-1α [42]. Also, SIRT3 expression in the liver is upregulated during fasting [13], most likely through mechanisms involving PGC-1α and ERRα [14]. Whether similar regulation takes place in diabetes is not known and requires further investigation.

Metformin is known to reduce PGC-1α induction by cAMP [43] and we recently showed that metformin also attenuates PGC-1α induction by glucagon [24]. Therefore, metformin is expected to downregulate expression of PGC-1α target genes, including ERRα. ERRα has been found to regulate the expression of SIRT3 [14] and, as in the case of SIRT3−/− mice, ERRα knockout is characterized by reduced ATP levels and lower tolerance to cold [44]. Therefore, it was hypothesized that downregulation of ERRα expression mediates metformin’s effect on SIRT3 regulation. Indeed, we observed that ERRα was upregulated by glucagon and that metformin reduced this response. In agreement with this result, ERRα siRNA significantly attenuated the PGC-1α-mediated induction of SIRT3. In addition, metformin reduced constitutive expression of ERRα and SIRT3 in primary hepatocytes and in the liver *in vivo*. However, dose response experiments in primary hepatocytes indicated that constitutive expression of SIRT3 was downregulated with lower concentrations of metformin than with ERRα, indicating that under these conditions ERRα does not play a major role in metformin-mediated downregulation of SIRT3. This is also supported by the experiments with ERRα siRNA, indicating that the reduction of ERRα expression did not affect constitutive SIRT3 expression. Therefore, regulation of SIRT3 expression by metformin is a rather complex event and involves other factors besides ERRα. Nevertheless, since the hepatic PGC-1α pathway is induced in models of insulin resistance and stimulates glucose production [41], repression of the PGC-1α-ERRα pathway is likely to play a significant role in reduction of SIRT3 expression by metformin in the diabetic liver.

Downregulation of SIRT3 is not mediated by AMPK. On the contrary, overexpression of AMPK induced SIRT3 expression. Recently it was shown that AMPK is activated by inhibition of mitochondrial respiration and not the reverse situation [45]. Therefore, downregulation of SIRT3 expression appears to be a primary response to AMPK activation and induction of SIRT3 by AMPK may represent a negative feedback loop.

Interestingly, one mitochondrial protein close to 17 kDa was strongly deacetylated by metformin treatment. The acetylation status of this protein is obviously regulated by an enzyme other than SIRT3. SIRT1 was upregulated by metformin and although SIRT1 has mainly nuclear and cytosolic localization, it has also been reported to reside in the mitochondria [46], [47]. It is, however, unclear if SIRT1 plays a role in the observed deacetylation.

Besides mitochondrial function, metformin was found to affect mitochondrial biogenesis. In agreement with previous findings using umbilical vein endothelial cells [36], metformin induced mitochondria biogenesis in cultured hepatic cells. This is probably related to the activation of AMPK by metformin since AMPK has been shown to be involved in mitochondrial biogenesis in the liver, and liver-specific AMPKα1α2−/− mice have reduced mitochondrial biogenesis [48], [49]. AMPK activation by metformin is mediated by a rise in the AMP/ATP ration due to the inhibition of mitochondrial respiration [45]. Therefore, activation of mitochondrial biogenesis by metformin can be seen as a compensatory mechanism for balancing repressed mitochondrial function. In agreement with this theory, metformin-induced inhibition of glucose production and the effect on the AMP/ATP ratio was found to be amplified in AMPK-deficient hepatocytes [3], [45].

In conclusion, we show that metformin downregulates expression of the mitochondrial deacetylase protein SIRT3 in the liver. This in turn alters mitochondrial acetylation status. Since SIRT3 is a key enzyme regulating mitochondrial function, including ATP production, we suggest that SIRT3 downregulation by metformin is involved in diminished ATP production after metformin treatment. Because suppression of ATP production appears to play a key role in the inhibition of hepatic glucose production, SIRT3 downregulation represents a novel pathway that may contribute to the therapeutic action of metformin.
